# *Cryptosporidium* spp. in wild rodents in the Poyang Lake region, China

**DOI:** 10.1051/parasite/2025056

**Published:** 2025-09-25

**Authors:** Qingqiu Zuo, Xiaodong Weng, Xu Wang, Hua Liu, Mingxiao Di, Xiaocheng Zhang, Bo Zhou, Chuizhao Xue, Ping Lu, Xiaoxue Peng, Yujuan Shen, Jianping Cao

**Affiliations:** 1 National Institute of Parasitic Diseases, Chinese Center for Disease Control and Prevention (Chinese Center for Tropical Diseases Research), NHC Key Laboratory of Parasite and Vector Biology, National Key Laboratory of Intelligent Tracking and Forecasting for Infectious Diseases, WHO Collaborating Centre for Tropical Diseases, National Center for International Research on Tropical Diseases Shanghai 200025 PR China; 2 Institute of Biological Resources, Jiangxi Academy of Sciences Nanchang 330096 PR China; 3 The School of Global Health, Chinese Center for Tropical Diseases Research, Shanghai Jiao Tong University School of Medicine Shanghai 200025 PR China

**Keywords:** *Cryptosporidium*, Zoonotic, Wild rodents, *Apodemus agrarius*, *Rattus losea*, Poyang Lake region

## Abstract

*Cryptosporidium* spp. are zoonotic protozoan parasites that cause diarrheal disease worldwide. Rodents can harbor diverse *Cryptosporidium* spp. and facilitate their transmission to the environment and other hosts, including humans. However, data on *Cryptosporidium* infection in wild rodents in the Poyang Lake region, China’s largest freshwater lake, remain scarce. Here, we investigated *Cryptosporidium* spp. in 273 wild rodents collected from seven sites adjacent to villages around Poyang Lake between 2022 and 2024. The rodents were identified by cytochrome c oxidase subunit I (COI) gene sequencing as *Apodemus agrarius* (*n* = 148) and *Rattus losea* (*n* = 125). Nested PCR targeting the small subunit ribosomal RNA (SSU rRNA) gene revealed an overall *Cryptosporidium* spp. infection rate of 16.5% (45/273, 95% CI: 12.3–21.9%), with 20.3% (30/148, 95% CI: 14.2–27.8%) in *A. agrarius* and 12.0% (15/125, 95% CI: 6.9–19.0%) in *R. losea*. Sequence and phylogenetic analyses identified seven *Cryptosporidium* species/genotypes: *C. apodemi*, *C. canis*, *C. muris*, *C. suis*, *C. ubiquitum*, rat genotype II, and rat genotype III. Notably, the detection of four zoonotic species (*C. canis*, *C. muris*, *C. suis*, and *C. ubiquitum*) highlights the potential risk of zoonotic transmission of *Cryptosporidium* spp. from wild rodents to humans in this region. These findings underscore the need for systematic surveillance and control of *Cryptosporidium* spp. in wild rodent communities around Poyang Lake.

## Abstract (fr)

Les *Cryptosporidium* spp. sont des parasites protozoaires zoonotiques responsables de maladies diarrhéiques dans le monde entier. Les rongeurs peuvent héberger diverses espèces de *Cryptosporidium* et faciliter leur transmission à l’environnement et à d’autres hôtes, dont l’homme. Cependant, les données sur l’infection à *Cryptosporidium* chez les rongeurs sauvages de la région du lac Poyang, le plus grand lac d’eau douce de Chine, restent rares. Nous avons étudié *Cryptosporidium* spp. chez 273 rongeurs sauvages collectés dans sept sites adjacents à des villages autour du lac Poyang entre 2022 et 2024. Les rongeurs ont été identifiés par séquençage du gène de la sous-unité I de la cytochrome c oxydase (COI) comme étant *Apodemus agrarius* (*n* = 148) et *Rattus losea* (*n* = 125). La PCR imbriquée ciblant le gène de l’ARN ribosomique de la petite sous-unité (ARNr SSU) a révélé un taux d’infection global par *Cryptosporidium* spp. de 16,5 % (45/273, IC à 95 % : 12,3-21,9 %), avec 20,3 % (30/148, IC à 95 % : 14,2-27,8 %) chez *A. agrarius* et 12,0 % (15/125, IC à 95 % : 6,9-19,0 %) chez *R. losea*. Les analyses de séquence et phylogénétiques ont identifié sept espèces/génotypes de *Cryptosporidium* : *C. apodemi*, *C. canis*, *C. muris*, *C. suis*, *C. ubiquitum*, génotype II du rat, génotype III du rat. Notamment, la détection de quatre espèces zoonotiques (*C. canis*, *C. muris*, *C. suis* et *C. ubiquitum*) met en évidence le risque potentiel de transmission zoonotique de *Cryptosporidium* spp. des rongeurs sauvages à l’homme dans cette région. Ces résultats soulignent la nécessité d’une surveillance et d’un contrôle systématiques de *Cryptosporidium* spp. dans les communautés de rongeurs sauvages autour du lac Poyang.

## Introduction

Cryptosporidiosis, a zoonotic enteric disease caused by *Cryptosporidium* spp. parasites, poses a major health risk to humans and animals worldwide [10]. Infections may occur through ingestion of *Cryptosporidium* oocysts *via* contaminated water and food, or direct contact with infected hosts [11]. This disease can cause severe and even life-threatening diarrheal illness in vulnerable populations, particularly in children and immunocompromised individuals [35]. To date, among the more than 170 species/genotypes of *Cryptosporidium* reported in various animals, 23 species and two genotypes are known to infect humans [12]. Understanding of the transmission dynamics and potential risks of *Cryptosporidium* spp. in populations in close contact with humans is key to developing effective prevention and control strategies.

Rodents, the most abundant mammals, are known for their adaptability and resilience, particularly because of their wide distribution near human settlements [3]. Many rodents are highly contagious carriers of zoonotic diseases, such as plague [1], hantavirus infection [36], and cryptosporidiosis [12]. To date, rodents have been confirmed to have been infected with more than 26 *Cryptosporidium* species and 59 genotypes. In particular, *C. hominis*, *C. parvum*, *C. meleagridis*, *C. felis*, *C. viatorum*, *C. mortiferum*, *C. muris*, *C. andersoni*, *C. suis*, *C. scrofarum*, *C. equi*, *C. erinacei*, *C. tyzzeri*, *C. occultus*, *C. ditrichi*, *C. wrairi*, and the skunk genotype are known to infect humans [12]. These diverse pathogens in rodents might pose a major zoonotic disease threat to the environment and to human populations.

A recent study has highlighted a substantial burden of human cryptosporidiosis in Jiangxi Province, where the total number of cases accounted for 13.95% (694/4975) of the national total, ranking second nationwide [43]. Furthermore, *Cryptosporidium* spp. infections have been documented in various farmed and companion animals, including bamboo rats (*Rhizomys sinensis*, 33.3%, 51/153) [23], cattle (*Bos taurus*, 12.8%, 71/556) [26], peafowls (*Pavo cristatus*, 9.52%, 10/105) [17], pigs (*Sus scrofa domesticus*, 4.0%, 41/1036) [42], and masked palm civets (*Paguma larvata*, 1.0%, 5/489) [46]. However, data on *Cryptosporidium* infections in wild animals remain relatively limited.

Poyang Lake (115°49′E–116°46′E, 28°24′N–29°46′N), the largest freshwater lake in China, is a critical source of drinking water for Jiangxi and neighboring provinces [20]. In recent years, an extensive and dense rodent community has been observed in the Poyang Lake region, which has been reported to have potential for a future rodent population outbreak [6]. These rodents are not only abundant in the lake region but also commonly found in agricultural areas and human settlements [3], thus posing potential public health concerns in sylvatic environments. The expansion of rodent populations might facilitate the transmission of pathogens, particularly waterborne pathogens. Therefore, investigating the presence and dynamics of *Cryptosporidium* spp. pathogens in the wild rodent communities within Poyang Lake’s ecosystem is crucial to safeguard public health, preserve ecological balance, and ensure sustainable development.

In this study, we aimed to investigate the infection status, genetic characteristics, and zoonotic potential of *Cryptosporidium* spp. in the wild rodent communities in the Poyang Lake region.

## Materials and methods

### Study area

**Figure 1:**
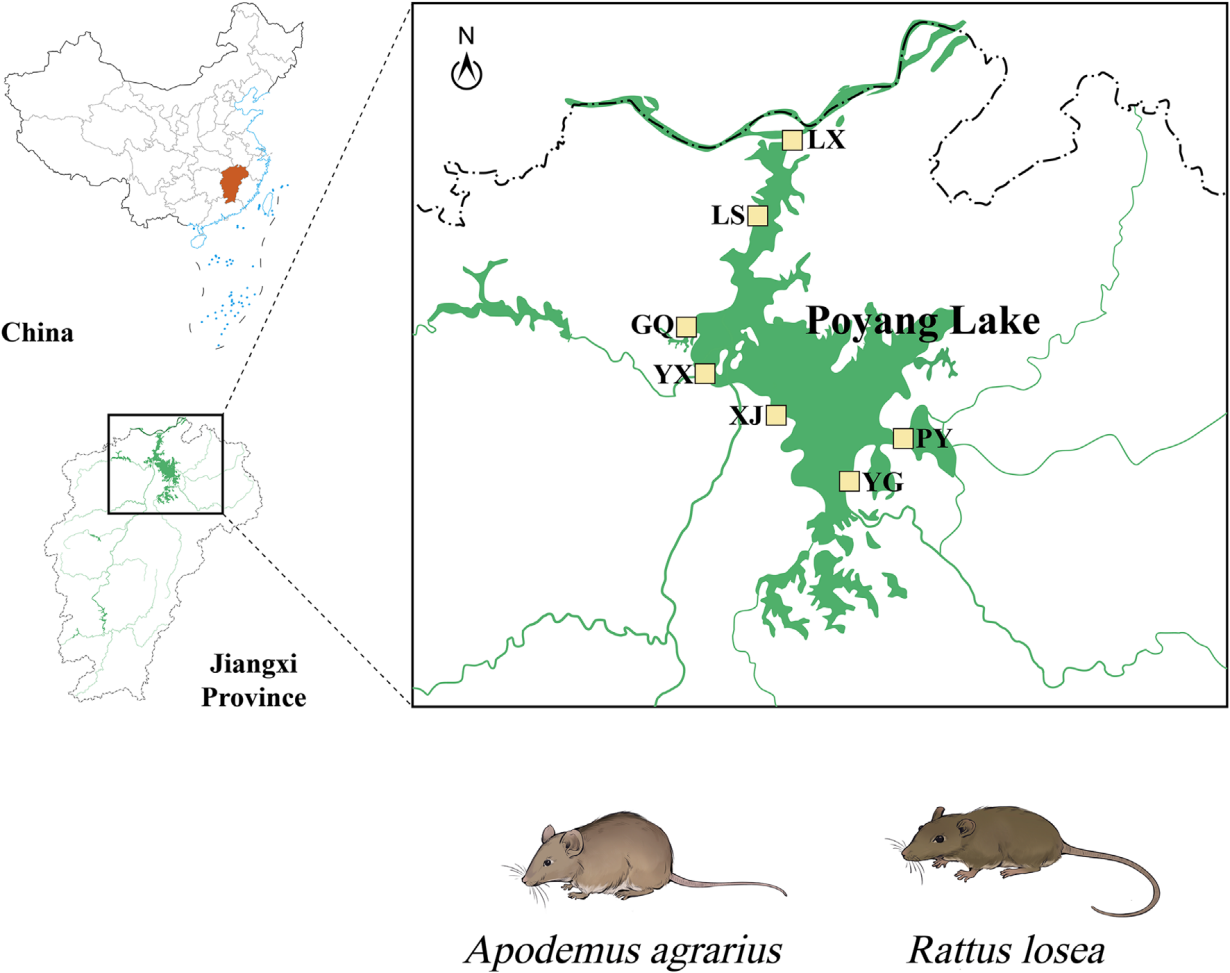
Study area and sampling sites (marked with yellow squares) around Poyang Lake, Jiangxi Province, China.

Field studies were conducted around Poyang Lake (28.24°N–29.46°N, 115.49°E–116.75°E), the largest freshwater lake in China, located in northern Jiangxi Province along the middle and lower reaches of the Yangtze River. In this region, the subtropical monsoon climate, with an annual average temperature of 17 °C to 18 °C, and the abundance of herbaceous vegetation, dominated by *Carex* spp. and *Phragmites* spp., provide suitable habitats for wild rodents, such as the striped field mouse (*Apodemus agrarius*) and the losea rat (*Rattus losea*). We selected seven sampling sites in farmlands and lakeside areas adjacent to both villages and water sources for wild rodent collection (Fig. 1 and Table 1).

**Table 1:** Information on *Cryptosporidium* spp. in wild rodents in the Poyang Lake region.

Category	No. examined	No. positive (%, 95% CI)	Species/Genotypes (No.)
*Host species* (*χ*^2^ = 3.03, *p* = 0.082)			
*A. agrarius*	148	30 (20.3, 14.2–27.8)	Rat genotype II (11), *C. muris* (4), *C. ubiquitum* (4), Rat genotype III (4), *C. apodemi* (3), *C. suis* (3), *C. canis* (1)
*R. losea*	125	15 (12.0, 6.9–19.0)	*C. apodemi* (9), Rat genotype II (2), *C. muris* (2), *C. ubiquitum* (1), *C. suis* (2)
			
*Sex* (*χ*^2^ = 0.60, *p* = 0.439)			
Male	142	26 (18.3, 12.3–25.6)	Rat genotype II (10), *C. apodemi* (9), *C. muris* (3), *C. suis* (3), *C. ubiquitum* (1), Rat genotype III (1),
Female	131	19 (14.5, 9.0–21.6)	*C. ubiquitum* (4), *C. muris* (3), Rat genotype II (3), Rat genotype III (3), *C. apodemi* (3), *C. suis* (2), *C. canis* (1)
*Age* (*χ*^2^ = 1.12, *p* = 0.772)			
Juvenile	17	2 (11.8, 1.5–36.4)	*C. ubiquitum* (1), *C. apodemi* (1)
Sub-adult	89	13 (14.6, 7.9–23.9)	*C. muris* (4), *C. apodemi* (4), Rat genotype II (3), *C. suis* (1), *C. ubiquitum* (1)
Adult	99	16 (16.2, 9.5–25.0)	Rat genotype II (7), *C. apodemi* (5), *C. suis* (4), *C. muris* (1)
Old	68	14 (20.6, 11.7–32.4)	Rat genotype III (4), *C. ubiquitum* (3), Rat genotype II (3), *C. apodemi* (2), *C. canis* (1), *C. muris* (1)
*Sampling year* (*χ*^2^ = 4.97, *p* = 0.083)			
2022	62	8 (12.9, 5.7–23.9)	*C. apodemi* (3), *C. muris* (2), *C. ubiquitum* (2), Rat genotype II (2)
2023	46	3 (6.5, 1.4–17.9)	Rat genotype III (2), *C. canis* (1)
2024	165	34 (20.6, 14.6–27.7)	Rat genotype II (11), *C. apodemi* (9), *C. suis* (5), *C. muris* (4), *C. ubiquitum* (3), Rat genotype III (2)
*Sampling site* (*χ*^2^ = 11.26, *p* = 0.081)			
YG	69	11 (15.9, 8.2–26.7)	Rat genotype II (3), *C. apodemi* (3), *C. ubiquitum* (2), *C. muris* (1), *C. suis* (1), Rat genotype III (1)
LX	56	7 (12.5, 5.2–24.1)	*C. apodemi* (2), Rat genotype III (2), *C. canis* (1), *C. muris* (1), *C. ubiquitum* (1),
XJ	48	13 (27.1, 15.3–41.8)	Rat genotype II (7), *C. suis* (3), *C. apodemi* (1), *C. muris* (1), Rat genotype III (1)
YX	37	5 (13.5, 4.5–28.8)	Rat genotype II (3), *C. ubiquitum* (1), *C. apodemi* (1), *C. muris* (1)
PY	33	9 (27.3, 13.3–45.5)	*C. apodemi* (4), *C. muris* (2), *C. ubiquitum* (1), *C. suis* (1)
GQ	21	0 (0)	/
LS	9	0 (0)	/
*Total*	273	45 (16.5, 12.3–21.4)	Rat genotype II (13), *C. apodemi* (12), *C. muris* (6), *C. suis* (5), *C. ubiquitum* (5), Rat genotype III (4), *C. canis* (1)

### Sample collection and preparation

This study involved 273 fecal samples from wild rodents, which were collected at seven sampling sites from 2022 to 2024 (Fig. 1, Table 1). Rodents were captured with snap traps (150 × 80 mm, Guixi Mousing Tool Factory, Jiangxi, China) baited with fresh sunflower seeds and then transported to the laboratory.

The sex of the rodents was determined through morphological observation of the genitalia. The rodents’ age was estimated according to body weight and used to divide the rodents into groups [19, 24]. *Apodemus agrarius* were divided into a juvenile group (body weight ≤ 16.0 g), sub-adult group (body weight = 16.1–23.0 g), adult group (body weight = 23.1–37.0 g), and old group (body weight > 37.0 g). *Rattus losea* were divided into a juvenile group (body weight ≤ 37.0 g), sub-adult group (body weight = 37.1–65.0 g), adult group (body weight = 65.1–92.0 g), and old group (body weight > 92.0 g). Fecal samples were placed in labeled 50 mL centrifuge tubes and stored separately at −20 °C before DNA extraction.

The protocol was approved by the Animal Experiment Ethics Committee of the Institute of Biological Resources, Jiangxi Academy of Sciences (No. 2022023).

### DNA extraction

Genomic DNA from ~200 mg rodent feces was extracted with a QIAamp DNA Stool Mini kit (Cat. #51604; QIAGEN, Hilden, Germany) according to the manufacturer’s instructions. The DNA was stored at −20 °C until PCR analysis.

### Host identification

A 750 bp fragment of the *mt*DNA cytochrome oxidase I (COI) gene was amplified with the primers BatL5310 and R6036R for rodent species identification. PCR conditions and primer design were as described by Robins *et al.* [33]. Each PCR reaction comprised 35 cycles of denaturation at 94 °C for 45 s, annealing at 54 °C for 60 s, and extension at 72 °C for 60 s. All amplified PCR products were visualized through 1% agarose gel electrophoresis. All successful amplifications were sent to Sangon Biotech (Shanghai) Co., Ltd. (Shanghai, China) for sequencing and then compared with entries in the NCBI database (http://www.ncbi.nlm.nih.gov/BLAST) for rodent species identification.

### Pathogen identification

*Cryptosporidium* species were determined with nested PCR and sequence analysis of the small subunit (SSU) rRNA gene [44]. Subsequently, the 60 kDa glycoprotein (*gp60*) gene was amplified with nested PCR to further subtype the detected zoonotic *Cryptosporidium* spp. (*i.e.*, *C. ubiquitum* [25], *C. suis* [22], and *C. canis* [21]). At least two replicates were included in the PCR analysis of each target for each sample. To control for contamination and ensure validity during the PCR process, we used *C. hominis* from patients with diarrhea as a positive control. RNase-free water was used as a negative control.

### Nucleotide sequencing and analysis

**Figure 2:**
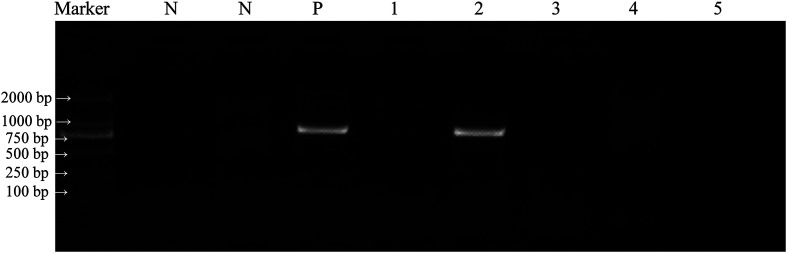
Representative agarose gel image showing PCR amplification products of the small subunit (SSU) rRNA genes of *Cryptosporidium* spp. (expected band size 830 bp). Marker: DL 2000 DNA marker; N: negative control; P: positive control; Lanes 1–5: samples.

All amplified PCR products were visualized through 1% agarose gel electrophoresis (Fig. 2). All successful amplifications were then sent to Sangon Biotech (Shanghai) Co., Ltd. (Shanghai, China) for bidirectional sequencing. Subsequently, all nucleotide sequences were analyzed in MEGA version 11.0.13 [39] and compared with the NCBI GenBank database (http://www.ncbi.nlm.nih.gov/) to infer species/genotypes/subtypes of *Cryptosporidium* spp.

### Phylogenetic analysis

To determine the phylogeny of the *Cryptosporidium* spp. identified in this study, we constructed maximum likelihood (ML) trees based on the SSU rRNA gene of *Cryptosporidium*. DNA sequences downloaded from NCBI GenBank and those obtained in this study were aligned in MEGA to construct the ML tree. The best-fit nucleotide substitution models were tested with jModelTest version 2.1.4 [31]. Finally, ML trees were constructed with MEGA with a “GTR + I + G” substitution model for *Cryptosporidium* species, with 1,000 bootstrap replications.

### Statistical analysis

The infection rates of *Cryptosporidium* spp. were determined according to the proportion of positive samples among the total rodent samples collected. The 95% confidence interval (CI) was used to quantify the precision of the positivity rate estimates. The chi-squared (*χ*^2^) test was used to statistically evaluate the variation in infection rates of *Cryptosporidium* spp. across rodent species, sexes, ages, sampling years, and sampling sites. All statistics were computed in R version 4.3.2 [32].

## Results

### Wild rodent community composition

A total of 273 wild rodents were captured from seven locations around Poyang Lake (Table 1). PCR and sequencing analysis of the COI gene identified two rodent species: *A. agrarius* (*n* = 125) and *R*. *losea* (*n* = 148) (Table 1). The rodent community was composed of four age groups: juvenile group (*n* = 17), sub-adult group (*n* = 89), adult group (*n* = 99), and old group (*n* = 68). The sex ratio of the community was balanced, with 142 males and 131 females (male-to-female ratio = 1.084, *χ*^*2*^ = 0.443, *p* = 0.506).

### Frequency of *Cryptosporidium* spp.

*Cryptosporidium* spp. DNA was detected in 45 of 273 fecal samples, with an infection rate of 16.5% (95% CI: 12.3–21.4%). The infection rate of *Cryptosporidium* spp. was 20.3% (30/148, 95% CI: 13.9–26.7%) in *A. agrarius* and 12.0% (15/125, 95% CI: 6.9–19.0%) in *R. losea*. Although no significant differences were observed by host species (*χ*^2^ = 3.03, *p* = 0.082), sex (*χ*^2^ = 0.60, *p* = 0.439), or age group (*χ*^2^ = 1.12, *p* = 0.772), the infection rate was higher in *A. agrarius* than *R. losea*, and higher in male than female rodents. Additionally, the positivity rate increased with rodent age (Table 1).

### *Cryptosporidium* species and genotypes

**Figure 3:**
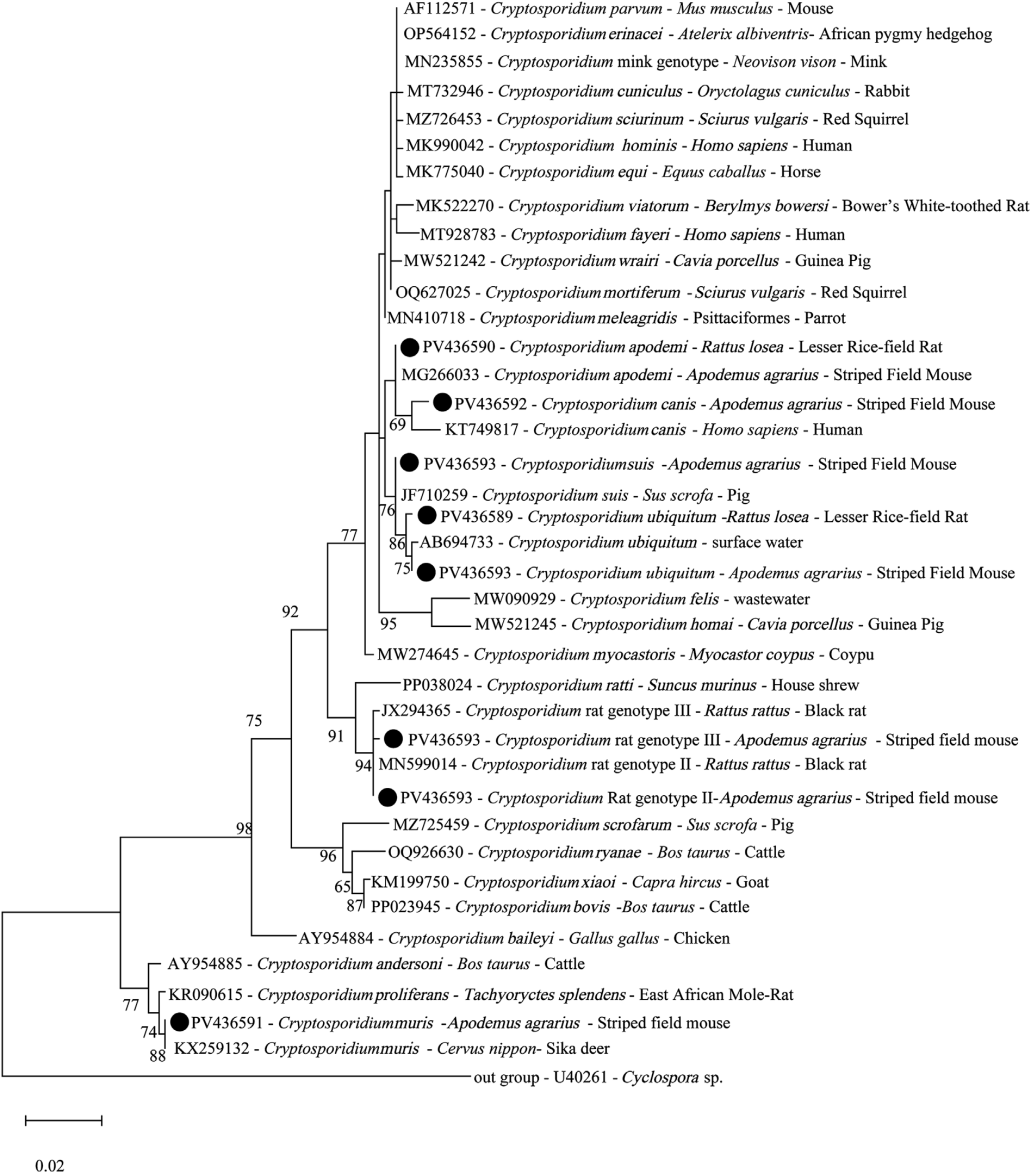
Phylogenetic analyses of the small subunit (SSU) rRNA genes of *Cryptosporidium* spp. with the maximum likelihood method (GTR + I + G model, 1000 replicates). Bootstrap values below 60% are not shown. *Cryptosporidium* species or genotypes identified in this study are highlighted with solid circles. Sequences are identified by their accession number, *Cryptosporidium* species, and host name.

Seven known *Cryptosporidium* species and genotypes were identified through sequence and phylogenetic analyses of the SSU rRNA gene: rat genotype II (*n* = 13), *C. apodemi* (*n* = 12), *C. muris* (*n* = 6), *C. suis* (*n* = 5), *C. ubiquitum* (*n* = 5), rat genotype III (*n* = 4), and *C. canis* (*n* = 1) (Table 1, Fig. 3). The *Cryptosporidium* species and genotypes identified in this study exhibited 98.6%–100% similarity with the GenBank reference sequences and were clustered accordingly (Fig. 3). The subtyping of *C. ubiquitum*, *C. suis*, and *C. canis* failed.

The distribution of *Cryptosporidium* spp. varied by rodent species, sex, age group, sampling year, and sampling site (Table 1). Analyses by rodent species indicated that rat genotype II was dominant in *A. agrarius*, whereas *C. apodemi* was dominant in *R. losea*. Analyses by sex indicated that rat genotype II and *C. ubiquitum* were dominant in male and female rodents, respectively. An assessment by rodent age indicated that *C. muris* and *C. apodemi* were dominant in the sub-adult group, whereas rat genotype II and rat genotype III were dominant in the adult and old groups, respectively. However, no dominant *Cryptosporidium* spp. was observed in the juvenile group, given the limited number of positive samples. An evaluation by sampling year indicated that *C. apodemi*, rat genotype III, and rat genotype II were most frequent in 2022, 2023, and 2024, respectively. An investigation by sampling site indicated that rat genotype II and *C. apodemi* were most frequent in YG; rat genotype III and *C. apodemi* were most frequent in LX; rat genotype II was most frequent in XJ and YX; and *C. apodemi* was most frequent in PY (Table 1).

### Nucleotide sequence accession numbers

All *Cryptosporidium* spp. gene sequences identified in this study have been submitted to the NCBI GenBank database under accession numbers PV436589–PV436596.

## Discussion

### High infection rate of *Cryptosporidium* spp.

As important reservoirs for *Cryptosporidium* spp., wild rodents substantially contribute to pathogen transmission in the environment and to humans. Understanding the infection status, genetic characteristics, and zoonotic potential of *Cryptosporidium* spp. in wild rodents is crucial for preventing and controlling local cryptosporidiosis outbreaks, particularly in water-abundant ecosystems. This study provides the first evidence of a high *Cryptosporidium* spp. infection rate (16.5%, 45/273) in a wild rodent community in the Poyang Lake region. This infection rate is lower than the 20.5% (3848/18804) reported in wild rodents worldwide [47], yet higher than the 9.78% (345/3526) reported in China [51]. The *Cryptosporidium* spp. infection rate in rodents exhibits substantial geographical variation globally. The highest infection rates have been documented in Europe (28.0%, 1,860/6,638), followed by Asia (18.6%, 1,394/7,510), North America (15.2%, 1,265/8,299), Oceania (13.7%, 56/410), South America (7.3%, 11/150), and Africa (2.2%, 3/135) [47].

In China, *Cryptosporidium* infections in rodents have been reported across 18 of 34 provincial-level administrative divisions [51]. Regional distribution patterns have revealed notably high infection rates in southeastern districts, including Hainan Province (50.0%, 75/150) [50], Fujian (18.2%, 6/33) [27], and Guangdong (18.0%, 21/117) [4], as well as in our study area around Poyang Lake in Jiangxi province. In contrast, lower infection rates have been reported in the central and western regions, such as Chongqing (3.6%, 4/111) [4], Shanxi (3.8%, 2/53) [30], and Gansu (2.5%, 10/399) [45]. Although a nationwide systematic assessment of *Cryptosporidium* in wild rodents in China is lacking, available data indicate distinct geographical patterns. A recent review has shown generally higher prevalence in southeastern than northwestern China [51]. This southeast-to-northwest decreasing prevalence gradient mirrors the trend observed in human cryptosporidiosis [43], thereby suggesting elevated overall transmission risk in these southeastern regions. The high prevalence observed in the Poyang Lake region, which was consistent with this broader geographical trend, might be partly explained by its unique environmental conditions. The warm, humid climate and extensive wetlands in this region enhance oocyst survival and facilitate waterborne transmission [20]. These favorable conditions are further compounded by host-specific factors. Abundant food from surrounding agriculture supports high rodent densities, which in turn accelerate pathogen transmission [6]. Finally, strong anthropogenic influence is likely to be a considerable contributing factor. The Poyang Lake basin is characterized by a dense human population and large-scale livestock farming. Our detection of multiple zoonotic *Cryptosporidium* species in this context provides direct evidence of active transmission chains involving wildlife, livestock, and humans. This confluence of factors establishes the region as a “One Health” hotspot requiring close public health monitoring.

Of course, prevalence is a complex trait influenced not only by broad ecological conditions but also by host characteristics, such as species, sex, and age [12]. In this study, although no statistically significant differences in *Cryptosporidium* positivity rates were observed among rodent groups, numerically higher infection rates were found in *A. agrarius* (20.3%, 30/148) than *R. losea* (12.0%, 15/125), and in males (18.3%, 26/142) than females (14.5%, 19/131); moreover, a gradually increasing trend was observed with age (Table 1). These findings highlight the need for systematic, large-scale surveillance of *Cryptosporidium* spp. in wild rodents in this region, combined with detailed analysis of their zoonotic risks.

### Zoonotic *Cryptosporidium* species

The need for surveillance is further underscored by our identification of four zoonotic species, *C. ubiquitum*, *C. muris*, *C. canis*, and *C. suis*, in these rodents, which might serve as reservoirs for human cryptosporidiosis. Among these, *C. ubiquitum*, which is frequently reported in humans [25], and is a geographically widespread species with a diverse range of mammalian hosts, including wild and domesticated ruminants, rodents, carnivores, and primates [14]. The role of rodents as an important reservoir for *C. ubiquitum* is highlighted by the identification of identical subtypes (XIIb and XIId) in rodents, humans, and the drinking water sources linking them, thus strongly suggesting a pathway for waterborne zoonotic transmission [25]. Our detection of *C. ubiquitum* in *A. agrarius* and *R. losea* – two dominant rodent species that frequently inhabit agricultural and residential areas – highlights a direct public health risk at the human-wildlife interface [6]. Similarly, the presence of *C. muris* in this study further reinforces wild rodents as important reservoirs. Although *C. muris* is known for its vast host range within rodent families, including Muridae, Cricetidae, Sciuridae, Caviidae, and Rhizomyidae [27], its importance as a human pathogen affecting primarily immunocompromised individuals in areas with poor sanitation cannot be overlooked [40, 47]. Despite being traditionally associated with domestic animals and humans [15], *C. canis* is increasingly being detected in wild canids [48, 41] and various small mammals [49]. Our detection of *C. canis* in *A. agrarius* and *R. losea*, in agreement with its discovery in other wild small mammals, adds substantial evidence indicating the sylvatic transmission cycle of *C. canis*. Given their ecological overlap with both wild and domestic canid populations, our findings suggest that these rodents might serve as potential bridge hosts, thus posing a transmission risk to domestic animals and humans. *Cryptosporidium suis*, a pathogen affecting primarily domestic pigs [34], has now been detected in diverse wildlife, including wild boar (*Sus scrofa*), red deer (*Cervus elaphus*), and red fox (*Vulpes vulpes*) [2, 7, 37]. Importantly, its presence in rodents is increasingly recognized, and it has been reported in species including the yellow-throated mouse (*A. flavicollis*) [7] and Brandt’s vole (*Lasiopodomys brandtii*) [16]. Our detection of *C. suis* in *A. agrarius* and *R. losea* further expands its potential rodent reservoir. However, the roles of rodents in the epidemiology of *C. suis* require careful interpretation. Distinguishing between mechanical carriage from a contaminated environment and a stable transmission cycle involving rodent-adapted subtypes is crucial for assessing zoonotic risk and warrants further investigation [12].

Our study also identified three dominant non-zoonotic species/genotypes in rodents: rat genotype II, rat genotype III, and *C. apodemi*. These species are largely considered specific to rodents: rat genotypes II and III infect primarily the house mouse (*Mus musculus*) [37], black rat (*R. rattus*) [18], and brown rat (*R. norvegicus*) [29], whereas *C. apodemi* is adapted to *Apodemus* spp*.* [5]. In this study, rat genotype II (*n* = 13) and *C. apodemi* (*n* = 12) were predominant in local wild rodents.

### Implications for public health in lake ecosystems

In this study, the detection of zoonotic species/genotypes, such as *C. suis*, *C. muris*, *C. canis*, and *C. ubiquitum*, in the wild rodent communities suggests a risk of pathogen transmission from wildlife reservoirs to humans. Egan *et al.* [12] have demonstrated that synanthropic rodents have a high prevalence of *Cryptosporidium* infection and consequently are likely to contaminate source water and wastewater. Similarly, the common occurrence of *Cryptosporidium* rat genotype IV and *C. muris* suggested that rodents are important sources of enteric pathogens detected in wastewater samples in Guangzhou, China [13]. Moreover, the detection of *C. muris* and *Cryptosporidium* rat genotype IV in Laguna Lake in the Philippines has revealed rodent fecal pollution of a lake system [9]. In another study in South Australia, *C. muris*, *C. ubiquitum*, and *C. tyzzeri* were among the dominant species detected in source water [38]. The risk of rodent-associated *Cryptosporidium* species and genotypes commonly identified in source water and wastewater should be carefully estimated. In particular, the Poyang Lake region is a critical ecological landscape and primary water source for human consumption, crop irrigation, and livestock husbandry. Therefore, determining the diversity of waterborne protozoan parasites and the host-parasite pathogen spectrum is critical for understanding parasite evolution and informing the development of effective prevention and control strategies.

Unfortunately, we were unable to subtype *Cryptosporidium* spp. by amplifying the *gp60* gene in the positive samples. For species with established *gp60* subtyping tools (*C. ubiquitum*, *C. canis*, and *C. suis*), our amplification attempts were unsuccessful. No specific products were yielded for *C. ubiquitum* and *C. canis*, whereas for *C. suis*, sequencing of amplicons revealed non-specific bacterial DNA. These outcomes suggested that these samples contained an extremely low parasite load insufficient for amplification of the single-copy *gp60* target gene. For other detected taxa, such as *C. apodemi* and the rat genotypes, standardized subtyping assays are not yet available. Future efforts should aim to clarify the zoonotic potential and transmission dynamics of *Cryptosporidium* spp. in this area. To overcome the issue of low-parasite-load samples, future studies might require more advanced methods, such as whole-genome amplification or targeted next-generation sequencing. Furthermore, a systematic investigation of a wider range of wild rodents should be conducted to more accurately estimate the prevalence and diversity of *Cryptosporidium* spp. in local wildlife.

## Conclusions

This study provides, to our knowledge, the first assessment of *Cryptosporidium* spp. in wild rodents around Poyang Lake, China. We observed a 16.5% overall infection rate in the wild rodent community composed of *A. agrarius* and *R. losea*. Seven *Cryptosporidium* species/genotypes were identified, including four zoonotic species (*C. muris*, *C. suis*, *C. ubiquitum*, and *C. canis*) and three rodent-adapted species/genotypes (rat genotype II, rat genotype III, and *C. apodemi*). These findings highlight the roles of wild rodents as reservoirs of *Cryptosporidium* diversity. The presence of zoonotic *Cryptosporidium* poses a potential public health risk to the local environment. Given the ecological importance of Poyang Lake as a crucial freshwater resource, systematic surveillance of *Cryptosporidium* in both rodents and their environment is essential for assessing the zoonotic transmission risks.
